# Sustainable application of biocides to promote hygiene and minimize antimicrobial resistance

**DOI:** 10.1093/sumbio/qvae015

**Published:** 2024-07-04

**Authors:** Thomas Willmott, Layali Jadaan, Gavin J Humphreys, Jian R Lu, Andrew J McBain, John Chapman

**Affiliations:** Division of Pharmacy and Optometry, School of Health Sciences, Faculty of Biology, Medicine and Health, The University of Manchester, M13 9PT, United Kingdom; Division of Pharmacy and Optometry, School of Health Sciences, Faculty of Biology, Medicine and Health, The University of Manchester, M13 9PT, United Kingdom; Division of Pharmacy and Optometry, School of Health Sciences, Faculty of Biology, Medicine and Health, The University of Manchester, M13 9PT, United Kingdom; Biological Physics Laboratory, Department of Physics and Astronomy, The University of Manchester, M13 9PL, United Kingdom; Division of Pharmacy and Optometry, School of Health Sciences, Faculty of Biology, Medicine and Health, The University of Manchester, M13 9PT, United Kingdom; Independent Researcher, Lincoln University, PA, United States

**Keywords:** biocides, antibiotic resistance, food preservation, sanitation and hygiene, AMR

## Abstract

Biocidal (microbicidal) products play a critical role in controlling microorganisms in healthcare, industrial, community, and home environments. There is, however, concern that their use and misuse might contribute to the evolution of antimicrobial resistance (AMR). When evaluating the risk associated with biocides, it is important to adopt an objective approach towards the evidence regarding both the benefits of their appropriate deployment as well as potential contribution to AMR. Biocide use should be restricted to applications where there are tangible benefits but also not unnecessarily restricted where genuine benefits can be demonstrated. From the perspective of sustainability, such benefits include the control and prevention of infections in clinical settings with associated reductions in antibiotic use, preservation of a range of products and materials, substantial reduction in infection risk for consumers, hygiene in the community (e.g. in public swimming baths), and microbial control in many facets of industry. Here, we will provide a critical assessment of the contribution of biocides to sustainability based on a critical evaluation of the literature, followed by offering our views on the future management of biocide use across the globe.

**Sustainability Statement**

Biocidal compounds (disinfectants, antiseptics, and preservatives) are essential actives in the control of microbial growth in medicine, industry, agriculture, and the domestic environment. Yet with the rise in antimicrobial resistance (AMR), there are concerns that their overuse will limit their efficacy and potentially contribute more broadly to AMR. Here, we discuss the global use of biocides and how their appropriate deployment can contribute to their long-term efficacy and sustainability in general. The aim is to promote responsible biocide usage to minimize environmental impact, promote human health, sustainable agriculture and food production, and improve food security. Appropriate use of biocides significantly reduces food wastage in the home and industry and may prevent infections, thus limiting the need for antibiotic use, a proven driver of AMR. Therefore, sustainable biocide use plays an important role in safeguarding human health and well-being, ensuring that biocides remain effective antimicrobial agents for future generations. This particularly relates to the United Nations Sustainable Development Goals good health and well-being (UN Sustainable Development Goal 3) and clean water and sanitation (UN Sustainable Development Goal 6).

## Introduction

**Figure 1.**
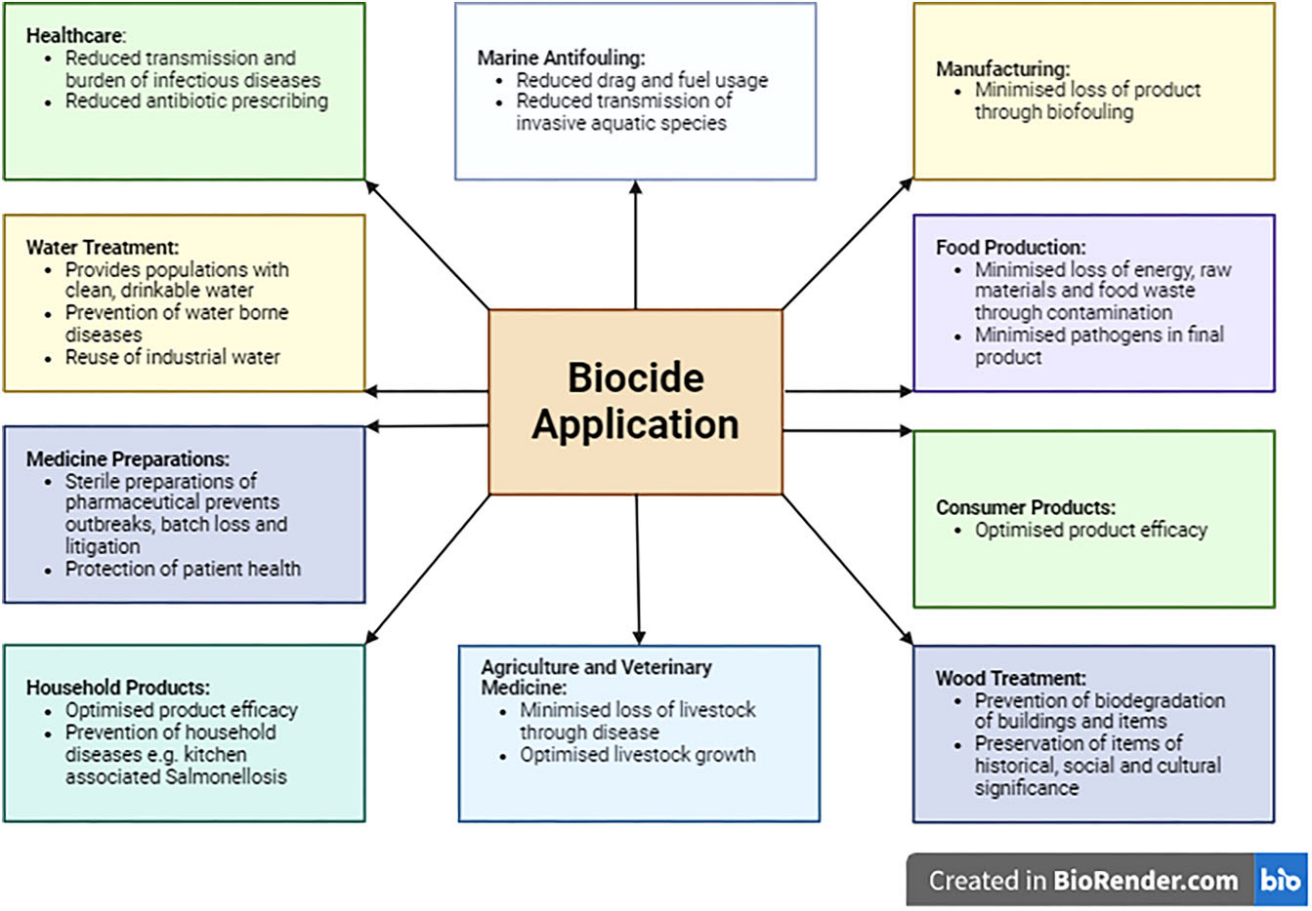
Benefits of biocide use in select applications. Biocides promote sustainability by preventing water, food, and contaminant diseases, product loss, and biofouling during manufacturing (created with BioRender.com).

Biocides are a diverse group of synthetic or semi-synthetic antimicrobial agents, that, above certain critical concentrations and under defined conditions, will kill living cells within specified times (Gilbert and McBain 2003). This includes microbicides, which are capable of killing microorganisms (including bacteria, yeasts, molds, viruses, and protozoa), and antiseptics, which are formulated agents that are safe for application to living things (Gilbert and McBain 2003). These compounds are often incorporated into disinfectants/sanitizers, which are formulations containing germicidal, microbicidal, or bactericidal agents that are safe for application to inanimate surfaces and that kill specified groups of disease-producing microorganisms (not including bacterial or fungal spores) (Gilbert and McBain 2003). These chemical compounds have been used effectively for centuries to control the colonization and growth of microorganisms. These products aim to stem the proliferation of organisms that are commonly implicated in infectious diseases, food spoilage and water contamination, industrial biofouling, and contamination in human and veterinary medicine (Maillard 2005). Consequently, targeted and effective biocide use is an appropriate measure to control microbial growth from the perspective of ethics and economics. This large range of applications is reflected by the wide spectrum of available biocide products (Fig. 1, Table 1).

**Table 1.** Examples of global biocide use.

Field	Application	Biocide(s) concentration	Reported benefits	References
Health-care	Surgical site infection	Chlorhexidine 2% + 70% isopropyl alcohol solution 2% chlorhexidine 2% chlorhexidine baths	Studies displayed reduced surgical infection rate that are better or similar to povidone-iodine application, which is more expensive and show side effect to neonatal patients Reduction in surgical site infection Reduced hospital acquired infection by 44.5% compared to regular soap bathing	Kucuker et al. (2023) Srinivas et al. (2015), Kapadia et al. (2016) Swan et al. (2016)
	Catheter associated infections	2% Chlorhexidine prepared in alcohol solution 0.1% Octenidine dihydrochloride prepared in alcohol Chlorhexidine and silver sulfadiazine catheter coating	The use of skin antiseptic resulted in lower frequency of local catheter associated infection cases compared to povidone iodine prepared with alcohol Reduced microbial skin colonization around catheter insertion site within 48 h that improves catheter associated blood stream infections Reduced catheter related blood stream infections rates compared to uncoated or povidone-iodine coated catheters	Guenezan et al. (2021) Lutz et al. (2016) Lorente et al. (2014), Paglialonga et al. (2014), Lorente (2016)
	Ventilator associated pneumonia	Single oral dose of 0.12% chlorhexidine	Reduction of early signs of pulmonary infections short term rates compared with usual intervention	Özçaka et al. (2012)
	Wound healing	Acetic acid 0.16%–0.31% Sodium hypochlorite 0.057% Polyhexamethylene biguanide 0.04%	Complete biofilm eradication in burn patient’s common clinical isolates *in vitro* exposure to below lethal concentrations within 3 h Reduction in patient’s microbial load promoting recovery of hard to heal wound Rapid decreased microbial load after 1 h of application to acute traumatic wound site	Halstead et al. (2015) Serena et al. (2022) Payne et al. (2018)
	Infection control	Hydrogen peroxide and chlorine dioxide	Water treatment to reduce hospital decontamination of *Legionella* spp. using biocides showed to be more effective than heat hyperthermia	Marchesi et al. (2016)
Food and agriculture	Food production	Benzalkonium chloride Peracetic acid and hydrogen peroxide Benzalkonium chloride, sodium hypochlorite, peracetic acid, and biguanide Benzalkonium chloride, glutaraldehyde, formaldehyde, and peroxyacetic acid	Applying biocides to prevent Listeria monocytogenes food contamination displayed high genetic diversity that was not associated with decrease tolerance to in use disinfectant concentrations nor to antibiotics Preventing spore forming molds through chemically Sterilizing food packaging machine and ventilators Effective antifungal properties on microorganisms that cause spoilage of bakery products Regular disinfection of animal housing and transportation controls the spread of infectious diseases without changes in antibiotics resistance or reduce effectiveness in pig farms	Roedel et al. (2019), He et al. (2022) Scaramuzza et al. (2020) Bernardi et al. (2019) Maertens et al. (2020)
Consumer products	Personal care	Piroctone olamine and zinc pyrithione Chlorine	Integrating biocides to anti-dandruff shampoo leads to healthier scalp and effective reduction in dermatophytes strains Regular swimming pools disinfection is critical in eliminating waterborne infections	Dos Santos and Dias-Souza (2017), Leong et al. (2019), Johnson et al. (2023) Papadopoulou et al. (2008), Rosende et al. (2020)

Biocides are regularly used across a wide range of fields to control the growth and eliminate microorganisms.

When applied appropriately, disinfectants, such as quaternary ammonium compounds (QUATs), have significantly reduced the risk of infectious diseases (Sato-Boku et al. 2019, Warren et al. 2022). For example, in hospitals, schools, and food processing plants disinfectants help restrict the transmission and persistence of contaminant microbes. Successful disinfectants eliminate pathogens and create a clean, hygienic environment, protecting individuals from risk of infection (Fox et al. 2022). Preservatives help keep products at their best, limiting negative effects on product aesthetics, product recalls, and ultimately the risks to the consumers associated with poor, contaminated, and degraded products. As populations grow and international travel becomes more common, the need for effective disinfectants has never been greater. QUAT-based products are approved by regulatory agencies around the world and are indispensable agents in modern hygiene. By appropriate use and formulating new, highly effective disinfectant formulations using QUATs and other active ingredients based on increased realism in biocide risk assessments we will help maintain an arsenal of biocidal agents to control harmful microorganisms for future generations (Fox et al. 2022).

### Biocide resistance and antibiotic cross resistance

Although the antimicrobial action of biocides has remained somewhat constant over the years, certain concerns have been raised (discussed in detail by Fox et al. 2022). Changes in microbial susceptibility towards biocidal products have been documented particularly based on studies in which the organisms are exposed under laboratory conditions (Gilbert and McBain 2001, Gilbert and McBain 2003), and the investigation of AMR to biocides tends to focus on a limited range of chemical types (Gilbert and McBain 2003). Most commercial biocidal products are usually available only as formulations with various excipients, which may alter and mask corrosiveness and odour, enhance stability, and reduce toxicity. For example, surfactants and sequestrants alter the biocide interaction with bacterial membranes and destabilize the cell envelope of microorganisms (Fox et al. 2022), which may result in enhanced antimicrobial activity (Cowley et al. 2015) and the mitigation of resistance (Forbes et al. 2017, Fox et al. 2022). Synergistic application of multiple compounds with intended or incidental antimicrobial activity can increase the activity spectrum of the formulated biocide and potentially minimize the risk of selection (Cowley et al. 2015). This chemical and formulation variety, in combination with different methodologies, makes *in vitro* studies of biocide resistance difficult to compare (Condell et al. 2012, Maillard et al. 2013, Gerba 2015, Jing et al. 2020). The major limitation of *in vitro* data that are often used in risk assessment is that they are arguably not reflective of the antimicrobial applications of biocides in real practice.

Studies examining changes in biocide susceptibility in healthcare and the community are scarce, with these studies reporting limitations in data interpretability (DeMarco et al. 2007, Ghanem and Haddadin 2018, Wesgate et al. 2019), notably due to differing definitions of resistance (as previously reviewed by Fox et al. 2022). Concern has been raised that extensive biocide applications could contribute to the decreased effectiveness of antibiotics, however, there have been conflicting reports linking biocides to antibiotic cross-resistance (reviewed by Oggioni et al. 2013 and Maillard 2018). A comprehensive report from an expert workshop that addressed the interconnection between microbial resistance to biocides and antibiotics concluded that even if each biocide represents a specific case, there is scientific evidence that biocides select for biocide resistance, but that there is, so far, no conclusive evidence that this also determines or will determine an increase in antibiotic resistance (Oggioni et al. 2013). Bacterial resistance mechanisms have been demonstrated to function by mechanisms that include efflux pumps, enzyme degradation, and various physiological and metabolic changes (as discussed in detail by Maillard 2018). However, during real life application, as opposed to *in vitro* investigations, in-use concentrations of biocides will most likely kill nosocomial pathogens before adaptation can occur (Fox et al. 2022). Overall, biocides positively impact hygiene and infection control and contribute to the sustainability of our antibiotic arsenal by reducing antibiotic use and resistance development (Uhari and Möttönen 1999, Azor-Martinez et al. 2018, Bronson-Lowe et al. 2018).

### Global biocide use

Global usage of biocides has increased over the years and is currently valued at 8.8 billion USD, with the biocide market predicted to grow 3.57% annually between 2023 and 2033 (Insights 2024). This is driven by the need to control microorganisms, regulatory requirements, and public awareness of hygienic benefits on overall health and quality of life. As a result, there is a consistent demand for use of biocidal products in home, healthcare, agricultural, and environmental settings (Kampf 2018, Jones and Joshi 2021). Biocides are a necessity for the protection on a homeowner facade to the hull of an ocean-going vessel. The considerable rise of AMR in healthcare and veterinary settings is an additional reason for increases in biocide deployment (Maillard and Denyer 2009). A focus on hygiene and antimicrobial stewardship has led regulators and governing bodies to examine protocols to appropriately manage the costs and benefits of global antimicrobial applications.

In healthcare, biocide application is critical to control microbial contamination and prevent outbreaks of hospital-acquired infections. Their use includes surface disinfection, device sterilization (McDonnell and Burke 2011), skin antisepsis, preservation of pharmaceutical preparations, and maintaining sterility in clean rooms (Meade et al. 2021). When implemented effectively, biocides have the potential to reduce infection rates and the need for antibiotics, thereby reducing the selective pressures that drive AMR (Scott and Bloomfield 2017). Hygienic practices have been associated with reduced infections and reduced antibiotic use (Krilov et al. 1996, Schmitz et al. 1998, Uhari and Möttönen 1999, Sandora et al. 2008, Ban et al. 2015, Azor-Martinez et al. 2018). The reduced spread of organisms with a low degree of pathogenicity may decrease the reservoir of resistance elements within a healthcare environment, limiting horizontal gene transfer to other pathogenic species (Bengtsson-Palme et al. 2018). Generally, biocides at in-use concentrations will effectively inactivate microorganisms, preventing any adaptation and potentially decreasing antimicrobial susceptibility. Therefore, proper use of biocides in healthcare is important for reduced patient mortality, morbidity, and AMR.

Biocidal compounds are used in marine transport to control contamination and fouling on the ship’s hull, which can occur during both storage and transit. Between 1999 and 2013, over 120 000 vessels with a combined hull surface area of approximately 571 km^2^ required protection (Moser et al. 2016). Marine biofouling compromises the efficiency of vessels and offshore infrastructure, which is a particular problem in the shipping industry (De Carvalho 2018). The increased drag force results in 35%–50% increased fuel consumption, which is directly related to greenhouse gas emissions (Schultz et al. 2011), raising the environmental footprint of the shipping industry. Therefore, periodic hull cleaning and reapplication with biocides are necessary to maintain consistent ship performance and reduce drag (Kim et al. 2024). Mechanical cleaning of hulls is time-consuming, has high labour costs, and is ineffective by itself. With new, stricter regulations on ship energy efficiencies, there is added importance on the protective biocidal hull coatings (Weber and Esmaeili 2023). An additional consideration is the transport of invasive species on contaminated hulls and in ballast water. Whilst there are concerns around possible bioaccumulation and biocide toxicity towards non-target species, their performance and cost effectiveness are unmatched. Their use in marine antifouling should be considered in the context of reducing energy expenditure and the prevention of invasive aquatic species being transported globally (Weber and Esmaeili 2023).

Biocides are applied to decrease manufacturing waste and preserve product integrity. Microbial contamination can significantly affect quality and safety throughout the manufacturing and retail processes. Microbes may be introduced via raw materials, processing equipment, water, and manufacturing personnel (Dao et al. 2018). From a sustainability viewpoint, product loss during manufacturing can be seen as a cumulative loss in resources that are used in production, including land, water, labour, money, and energy (Corona et al. 2019). This is of note in the food industry, where ∼40% of food loss occurs at the production, processing, storage, and transportation levels (UNEP 2021). Integrating biocides allows products to meet safety requirements in food and beverage production. Sterility is of particular importance in the preparation of formulated foods for children. Surface active biocidal compounds are commonly used as chemical disinfectants to ensure microorganisms do not remain on equipment and surfaces and form biofilms (Jones and Joshi 2021). Biocides help facilitate high-volume food production with consumers demanding healthy, minimally processed, nutritious food with minimal additives in the final products (Condell et al. 2012). All manufacturing processes that use water will require biocides. Therefore, by limiting microbial contamination, biocides promote sustainable, efficient, and productive manufacturing with minimal spoilage and product loss.

In pharmaceutical manufacturing, biocidal products are paramount in reducing product contamination. Sterility of medicines is of absolute importance, with microbial contamination presenting a risk to patients and consumer life quality, and contamination of parenteral products possibility having severe implications. In 2012, there was a fungal meningitis epidemic from contaminated injectable medicine across 23 US states, causing 751 cases and 64 deaths, and numerous associated legal challenges (Abbas et al. 2016). Disinfection of surfaces and equipment and patient skin disinfection are all required to decrease the risk of microbial contamination during the preparation and administration of medicines (Suvikas-Peltonen et al. 2017). Clean rooms to produce and formulate the drugs are a necessity, and biocides are required to maintain these aseptic environments. A wide range of biocidal formulations that are used in the preparation and administration of pharmaceuticals. This ensures and maintains sterility, protecting consumer health, and reducing product loss.

Many household products are water-based and, as such, are particularly susceptible to microbiological contamination. Biocidal products are effective at reducing pathogens in domestic environments (Jones and Joshi 2021). Biocides are used for home infection control, with poor home hygiene being proposed to be a factor in the transmission of community-based infections (Scott 2013). Several studies at home and in everyday life situations demonstrate the transmission of pathogens on hands and surfaces in numbers sufficient to cause infections (Scott and Bloomfield 2017). Biocidal compounds such as triclosan, hypochlorite, and chlorine-based disinfectants are essential to minimize microbial contamination of regular household products such as shampoo (Alfhili and Lee 2019), toilet disinfectants (Abney et al. 2021), and public leisure areas including swimming pools (Borgmann-Strahsen 2003). The risk of domestic transmission of pathogens and food-borne pathogens such as *Salmonella* and *Campylobacter* warrants the home use of biocides as well as their use as preservatives in many domiciliary products. In the household arena, preservatives extend product life, reduce waste, and enable a healthier environment, whilst antiseptics are important in primary infection control.

Biocides are relied on in animal husbandry to prevent animal infections, help farmers cut losses, and boost productivity with crop protection and pest control. Biocides are applied regularly in livestock farming to prevent zoonotic infections and to maintain sterility in food processing and crops (Davies and Wales 2019, Guo et al. 2021). The UK foot and mouth outbreak in 2001 cost over £8 billion, with 6.5 million animals culled, drastically affecting the country’s agriculture and food security (Anderson 2002, Scudamore and Harris 2002). Disinfectants are important in the elimination of viruses in livestock barns and roads (Kim et al. 2013), although little research has been published in the area (Robinson et al. 2016). The US porcine epidemic diarrhoea virus emerged in May 2013 (Huang et al. 2013), resulting in an epidemic that is estimated to have cost between $900 million and $1.8 billion (Paarlberg 2014). A combination of quaternary ammonium and glutaraldehyde disinfectants has proven to be effective against porcine epidermic diarrhoea virus (Holtkamp et al. 2016). Disinfection of agricultural premises helps reduce outbreaks, which would be detrimental to food production and food security (Condell et al. 2012). Meat production stages are often geographically segregated, which requires animals to be moved through different farms throughout their lifetime, which presents vulnerabilities to naïve populations to novel and emerging pathogens. Applied appropriately, biocides will reduce the need for culling animals and limit the likelihood of subsequent outbreaks. This will help to end hunger, achieve food security, improve nutrition, and promote sustainable agriculture.

Clean water is a sustainable resource when appropriately managed. Biocides play a major role in maintaining the microbial quality of municipal and industrial water, amounting to a 4.7 Bn USD global market in 2024 (Aceanalytic 2018). The largest applications of biocides in water treatment are in municipal (drinking water), the oil and gas industry, power plants, and pulp and paper production. Growth in municipal water application is driven by population growth, urbanization, degradation of water sources, and compliance with regulatory standards (Markets 2019). Provision of clean drinking water allows humans to congregate in cities and maintain a healthy and productive population. Simple disinfection technologies (like bleach) provide rural populations with clean water as well, allowing them a sustainable existence. Microbially clean drinking water has a major role in ensuring a healthy, productive, and sustainable population. Biocides ensure longevity and sustainable management of clean water and sanitation for all populations.

There are many applications of biocides in industrial water treatment (Laopaiboon et al. 2001, Bartlett and Kramer 2011, Kéki et al. 2019) but the goal of each is maintaining efficient production of clean products, preventing equipment damage and system fouling, public safety, and regulatory compliance. Biocides can inactivate most bacteria and algae without having strong detrimental environmental effects (Gregg and Hallegraeff 2007). A prime factor in efficient production is the ability to use a resource to its maximum, and water is a key resource. In cooling towers, biocide treatment reduces biofilm growth and biofouling, thus increasing the deployment time between shutdowns and cleaning, an expensive and time-consuming endeavour (Pereira et al. 2017, Di Pippo et al. 2018). The pulp and paper industry provides another example of the impact of biocides on sustainability (Lindholm-Lehto et al. 2015). Mills use a great deal of water and operate in a ‘once-through’ manner; i.e. water enters the mill, is used once, and is then returned to the river. The current trend is for mills to move to recycling systems in which the water is re-used many times, which can be achieved with the aid of biocides and other water treatment chemicals. This reduces water use, pollution, and improves the quality of the surrounding environmental waters. Although there can be low levels of microbicides in the agricultural water system, most agricultural water is untreated groundwater (Chen et al. 2018). Biocides, therefore, have a significant role in the sustainability of municipal and industrial waters. Treatment of industrial waters permits the recycling of water and associated reductions in pollution, reduces stress on local water sources, and contributes to the sustainable production of goods.

Due to the potential harm to the environment, there are concerns about contaminant biocides in the waterways and soils. Whilst new biocides to prevent marine biofouling are intended to be environmentally less harmful compared to older biocides, the problem of toxicity on several marine species remains (Amara et al. 2018). Water disinfection is required to control waterborne diseases, yet toxic disinfection by-products (DBPs) have been identified, although only <20 are currently governmentally regulated (Plewa and Richardson 2017). Disinfection using ozone or peracetic acid is also a critical method used to inactivate pathogenic organisms during wastewater treatment to minimize ecological and health risks before discharge or reuse (Morrison et al. 2022, Lin et al. 2024). The aquatic toxicity information of DBPs is still limited. Much needs to be studied regarding the potential impact of biocidal mixtures and subsequent degradation products that could affect human and aquatic life. A new, interdisciplinary, integrated approach is required to assess these risks.

Biocides have an important role as a control method against microbial fouling and biodeterioration of building materials (Koziróg et al. 2016, Rajkowska et al. 2016). Wood has been used in the construction of houses, ships, weapons, and tools for centuries, and due to its chemical composition, this natural organic material is readily colonized and degraded by microorganisms and insects. Historically, creosote (a hydrocarbon derived from coal tar), oil-borne pentachlorophenol, and water-borne arsenicals were used (Schultz et al. 2007), and in modern times, microbicides including QUATs such as benzalkonium chloride and hydrogen peroxide are used (Jurado et al. 2010, Sterflinger and Piñar 2013, Koziróg et al. 2016). For example, Nowicka-Krawczyk et al. (2019) found that QUAT-containing biocides were successful antifouling agents for protecting historical materials against biodeterioration by phototrophs. Objects of cultural, architectural, infrastructure, or social importance must have their appearance and condition maintained by controlling the colonization and growth of microorganisms on wood and stone (Geweely 2023). By preventing biodeterioration, it is possible to extend the lifetime of many sites or important objects, allowing future generations to witness and study historical and cultural items across the globe.

### Sustainable production, use, and disposal of biocides

Biocides are essential for maintaining the health, food security, and economics of the current generation. Their sustainable and responsible use is therefore required to control microorganisms and mitigate the adverse effects of microbial proliferation for future generations. Current and future biocides need to be evaluated from conception, manufacture, and global application to balance their vital role in modern microbiology with long-term risks and negative effects. Preventative measures, training and education, in-depth phenotypic and genomic analyses of resistance, and global monitoring and reporting of resistance all need to be considered by basic scientists, pharmaceutical manufacturers, and healthcare professionals to ensure and maximize sustained biocide efficacy. Biocides must be regulated through life cycle assessment to examine sustainability at all stages of their production, use and disposal. This ‘cradle to grave’ concept needs to include resources, material processing, production manufacturing, distribution, use, and disposal of biocides (ISO 2006a,2006b). Raw materials and chemical processing should be quantified for energy and environmental costs. Optimal manufacturing guidelines need to be constructed to ensure minimal wastage and excretion of harmful products into the local water environment. An example of life cycle assessment is that of commercial antifouling paints in the aquatic environment (Rossini et al. 2019).

### Development of new agents and formulation

The needs of manufacturers are often better served by (re)formulating approved biocidal substances rather than developing new biocides. Financially, the cost of registering a new biocide is significantly higher than adjusting an approved biocide, so often development is based on formulation of pre-existing agents. Another important factor is approval time, with formulation products usually taking a year for a decision, whilst a newly developed biocide can take up to 3 years and 10 years under review. In some formulated products, the active biocidal substance can be at much lower concentration, which lead to concerns that it might drive selection for adaptive population (Gerba 2015). However, formulated products also require comprehensive risk assessment, including environmental studies, for market approval (Lee et al. 2023). Yet, there is still ongoing research into new biocidal agents. For example, an *in vitro* study explored conjugating essential oils with nanoparticles against a few crop pathogens, and the effective antimicrobial activity displayed can be explored to replace chemical pesticides (Bravo Cadena et al. 2018). New agents and formulations are required to continue to provide end users with a range of biocides to optimally control microbial growth and maintain the sustainable outcomes provided by biocides.

### Optimizing risk assessments and best practice codes

Currently, the majority of studies reporting reductions in biocide susceptibility have used active compounds in aqueous solutions (Walsh et al. 2003, Cottell et al. 2009, Forbes et al. 2014, Alonso-Calleja et al. 2015, Maillard 2018). Whilst providing insights into mechanisms of biocide resistance, biocides are mostly deployed in formulated products with multiple additives that enhance potency and at much higher working concentrations (Cowley et al. 2015, Knapp et al. 2015). This highlights the need for risk assessments that more accurately reflect the way in which biocides are deployed by end users. Therefore, the development of realism-based approaches and comprehensive risk assessments will inform the best practice for applying biocides to control microbial growth (Forbes et al. 2019, Fox et al. 2022). A general standard criterion must be established to allow comparisons between biocidal products to cover all aspects, including efficaciousness, human health, and environmental effects. This would increase our understanding of reduced susceptibility and optimize biocide usage, prolonging their efficacy. These criteria need to be assessed with respect to risks to human health and environment, considering effects on the ecosystem and biodiversity following disposal. In Europe, the disposal of biocides is controlled under the European Biocidal Product Regulation (EU 528/112) (EU 2012). Any hazardous environmental properties need to be mitigated and communicated to the user via product labelling and safety data sheets. As biocidal products are routinely detected in environmental samples (Pedrouzo et al. 2009, Wilson et al. 2009, Kumar et al. 2010), environmental release of antimicrobial substances needs to be assessed and controlled globally.

### Regulation of sales and reducing overall biocide usage

The advertising of biocidal products must be approved by the relevant scientific regulatory bodies (e.g. the environmental protection agency and the biocidal products regulation) to ensure messages are accurate scientific claims without false impressions. Such regulation should be applied to biocide sales, ensuring biocides are only used by appropriate user groups and are utilized in appropriate quantities. This process will also provide opportunities to raise awareness for possible risks and the safe use of biocides and facilitate optimization. This will also allow opportunities to educate users on additional information such as preventative measures for reduced susceptibility or biocide-free substitutes to promote sustainability (Development 2023). An example is using biocides to sterilize medical devices instead of single-use devices (Rowan et al. 2023). Limiting single use devices reduces medical waste, financial costs and carbon emissions from production (MacNeill et al. 2020).

### Licencing of biocides

Significant efforts have been made to regulate biocide production through specialized committees (Carnesecchi et al. 2023, Lee et al. 2023). These aim to ensure the safe distribution and production of chemicals. Comprehensive risk assessment sheets are required for pre-market approval, which allows the end users to properly evaluate the positives and negatives of biocide applications (Elekhnawy et al. 2020). Afterwards, new compounds must be monitored to appropriate safety standards. All products must include a safety sheet that states chemical characteristics, storage, and stability under different conditions with clear labelling (Oelkers 2021). Microorganism adaptation studies, short-term and chronic mammal toxicity exposure studies must be conducted following the testing guidelines provided (Scholz et al. 2013). Furthermore, environmental regulation risk assessment studies are required before biocide authorization, including hazard identification, risk characterization, biodegradability, effect, and exposure assessment of the biocide against multiple life forms and natural waters (Moermond et al. 2012, van Dijk et al. 2021). This should include laboratory-based studies utilizing statistical modules that predict the risks of active substances in the environment (Hommen et al. 2010). The same biocidal product can be subjected to different approval processes depending on intended use, such as cosmetics preservatives, where no environmental studies are needed (Kättström et al. 2022).

### Training and education: information awareness

It is important for biocide end users to understand the impact of improper biocide application. Obligatory training programmes and further education of professionals can help disseminate and enforce this information. To reach non-professional users, product labelling and packaging must be clear and convey appropriate messages to help with information and awareness raising. Additional communication pathways could also be considered, such as websites and smartphone apps. Independent consultants with a science-based background could be utilized to help inform professionals and non-professionals on how to treat harmful microorganisms in the most sustainable efficient ways. Many lessons can be learned and parallels drawn from the antimicrobial stewardship programmes that were rolled out in response to rapidly rising antibiotic resistance. These programmes highlighted the necessary role of antimicrobial resistance education in the training of healthcare professionals as part of a move to a more sustainable future (Gyssens 2018, Septimus 2018, Satterfield et al. 2020, Monmaturapoj et al. 2021).

### The risk of biocide use in sensitive/high-risk areas

Sensitive, protected areas exist worldwide. Biocides can be used both directly and indirectly in these areas. Chemical pollutants can cause toxicity to aquatic environments, leading to the loss of habitat, and biodiversity and are a threat to human health. There is still a need to evaluate the hazard of biocides and their metabolites for aquatic environments, with scarce data available for many of these compounds (Hernández-Moreno et al. 2019). Globally, there are hot spots for AMR (Collaborators 2022, Zhou et al. 2022), and there are many socio-economic and epidemiological factors that contribute to these areas of high risk. For example, areas that emit pharmaceutical wastewater containing high levels of antimicrobials have been shown to introduce these compounds into the surrounding area (Zhang et al. 2015, Zheng et al. 2021). Chemical precursors and end product biocides used in manufacturing and application eventually find their way to the environment (SCENIHR 2009). High levels of triclosan have been identified in river and wastewater effluents from wastewater treatment plants (Wilson et al. 2009, Kumar et al. 2010). Studies of environmental isolates from areas where antimicrobials are heavily used have shown a high prevalence of efflux genes and a decreased susceptibility to biocides (Liu et al. 2015, Grande Burgos et al. 2016, Hijazi et al. 2016). The existence of these sensitive areas and regions of high AMR needs to be considered appropriately in biocide risk assessment in addition to the treatment of wastewater from biocide and antibiotic manufacturers (Chu et al. 2021, Wang and Chen 2022).

Measures can be taken to minimize risk to aquatic life in highly sensitive areas. It is important that there is sufficient knowledge about possible negative impacts in as many environmental scenarios during its pre-market approval process. Environmental surveillance is proposed to study the potential spread and occurrence of biocides, both globally and locally in sensitive areas (Munk et al. 2022, Development 2023). This requires the continuous development of assessment methodologies across disciplines. Preventative measures or biocide-free substitutes should be considered in such sensitive areas. For example, leisure boats that have biocides incorporated into the hull paint should be avoided in sensitive areas (Development 2023). Biocides that are more environmentally friendly should be selected, such as hydrogen peroxide, as it only degrades into water and hydrogen (Jones and Joshi 2021).

### AMR monitoring and surveillance

**Figure 2.**
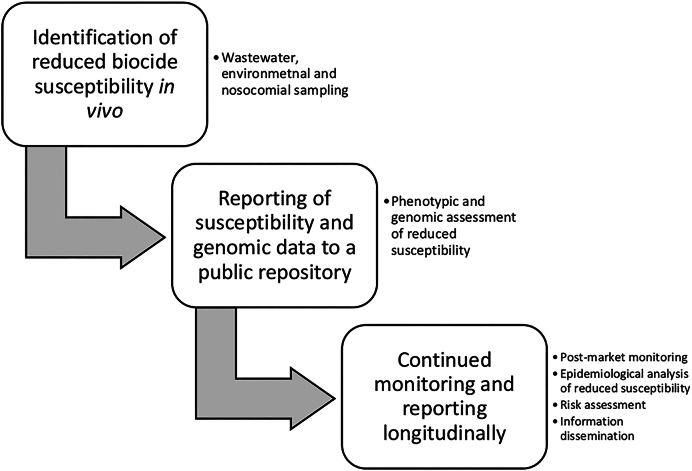
Flow chart for reporting reduced biocide susceptibility. By uploading an incident of reduced susceptibility to public, global repository, microbiologists can freely monitor and analyse risk rapidly. This would eliminate the reliance on delayed scientific publications and inaccess to datasets.

Whilst there has been a push for global reporting and systemic analysis of antibiotic resistance (Sugianli et al. 2021, Ayobami et al. 2022, Collaborators 2022, Okolie et al. 2022), no such system exists for biocides. There is a need for environmental, healthcare, and industrial monitoring of biocide resistance, which identifies the risks not detected during risk assessment (Fig. 2). Such a system would require international cooperation and standardization of reporting when there is currently a lack of clear definitions hindering comparative analysis of biocide resistance (Fox et al. 2022). Little evidence exists presently on what types of bacteria and resistance mechanisms best predict reduced susceptibility, ARGs and the overall abundance of resistant bacteria and diversity existing in a given environment (Bengtsson-Palme 2018, Bengtsson-Palme et al. 2023). Currently, there are no guidelines for monitoring the use of disinfectants on a large scale in terms of environmental safety (Nabi et al. 2020). Furthermore, once biocides have been licensed and marketed, post-market monitoring and surveillance can be beneficial in certain circumstances.

### Enforcing proper use

Once identified, adherence to risk control measures and best practice codes that aim to optimize the use of biocides will be critical to ensuring success. Authorities would have to monitor end users and the global biocide market to guarantee compliance. These could be governmental regulators, such as Public Health England or the Department for Environment, Food and Rural Affairs. Certification of recognized training programmes and auditing schemes could help promote fulfilment to sustainable practices and could even be a selling point to customers, auditors, and regulators (Development 2023), encouraging compliance. Completion of these courses and training programmes, and the addition of biocide risk assessments to local health and documentation, could be made mandatory. Concerning the commercialization of biocidal products, controls must then be required to prevent inappropriate advertisements and false information surrounding biocide use. This would require the establishment of an adequate global regulatory body with a standard set of guidelines that is capable for enforcement. Currently, such a body does not exist.

## Conclusion

Biocides are essential to prevent and control microbial contamination in healthcare and industrial environments. Like any individual agent, sub-lethal exposure of microorganisms to biocides may induce a bacterial response that favours reduced susceptibility (Fox et al. 2022). Yet the application of biocides at in-use concentrations will generally inactivate the microorganisms present at the site of application preventing any adaptation. Current risk assessment of biocides based on sub-lethal exposure of laboratory strains may be misinforming regulators, manufacturers and end-users. Ultimately, a greater understanding of the outcome of microbial exposure to biocides will promote their safe application, and the development of effective and safe biocidal products.

Here, we have put forward a framework to help ensure the efficacy of biocides is maintained. This includes accurate *in vivo* reporting of reduced susceptibility, complemented with phenotypic and genomic analyses of reduced susceptibility using appropriates that mimic real like applications. Governing and regulatory bodies need to ensure biocides are sold and applied appropriately to ensure any risks to the environment and human health are mitigated. We suggest that the appropriate and targeted use of biocides does have a role to play in sustainability. It is critical to move towards more sustainable, targeted applications of biocides, supplemented with a strong background of research into resistance mechanisms and epidemiology. This will help to ensure that biocidal products are maintained as an effective and efficient source of antimicrobial control and that antibiotic efficacy is maintained for future generations.

## Data Availability

As a review article, there are no data to make available.
